# Bilateral Metachronous Testicular Chloroma: A Rare Clinical Entity

**DOI:** 10.5812/numonthly.12021

**Published:** 2013-11-13

**Authors:** Athanasios Dellis, Dimitrios Boutsis, Evangelos Spyropoulos, Ioannis Galanakis, Angelos Panagopoulos, Athanasios Papatsoris

**Affiliations:** 1 Department of Urology, Naval and Veterans Hospital, Athens, Greece; 2 Department of Haematology, Naval and Veterans Hospital, Athens, Greece; 3 University Department of Urology, Sismanoglio Hospital, Athens, Greece

**Keywords:** Sarcoma, Myeloid, Testis, Leukemia

## Abstract

Testicular chloroma is an unusual form of extramedullary acute myeloid leukemia. We present a rare case that after chemotherapy relapsed with the appearance of metachronous testicular chloroma and we suggest prophylactic radiotherapy.

## 1. Introduction

Chloroma is a rare extramedullary neoplasm composed of immature myeloid cells (myeloid sarcoma) and the term is attributed to the green color attributed to myeloperoxidase (1, 2). The disease usually presents in patients with a history of acute myeloid leukemia (AML) and there are a few cases with localization of chloroma in one of the testises (3). We report an extremely rare case of bilateral metachonous chloroma of the testis.

## 2. Case Report

A 70-year-old patient presented with a six-month history of painless right scrotal swelling that was getting progressively worse the last weeks. The initial general practitioners’ diagnosis was epididymitis due to the relevant symptoms and signs. The patient received antibiotics for six months and anti-inflammatory drugs for several weeks, with no obvious relief. The ultrasound (US) revealed an edematous and heterogeneous right testis as well as hydrocele. A scrotal magnetic resonance imaging (MRI) confirmed the US findings of heterogeneity and following paramagnetic substance infusion, no localized enhanced testicular parenchymal lesion was detected. The contralateral left testis was normal on both the US and MRI tests. The patient’s chest X-ray and the testicular tumor marker levels were within the normal range.

**Figure 1.**
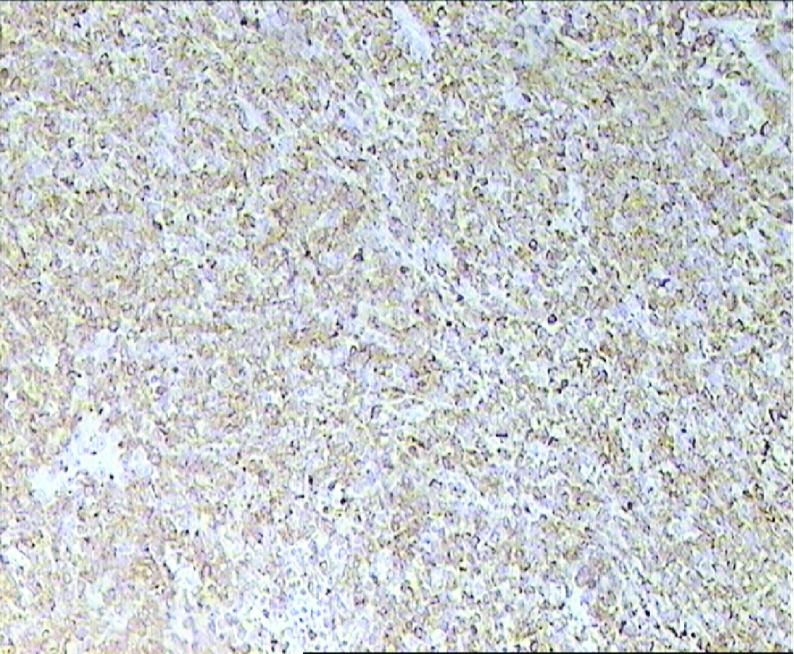
Chloroma of the Testis, Myeloperoxidase Staining (x100)

**Figure 2.**
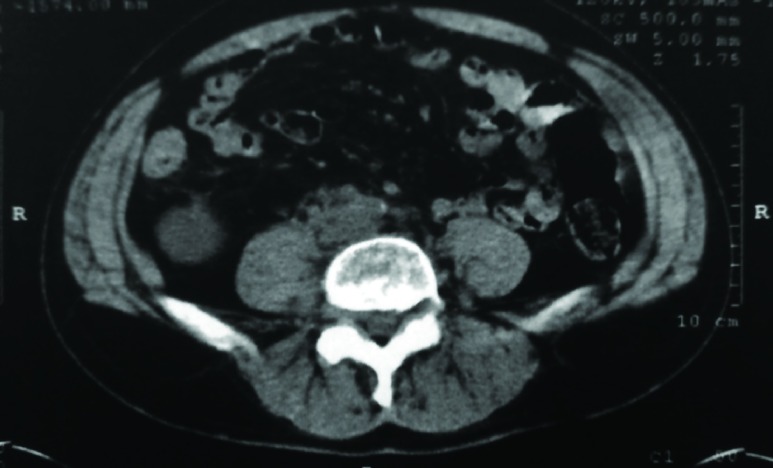
Solid Mass (Arrow) in the Right Paraaortic Area

Due to the abovementioned imaging findings, the patient underwent a right radical orchiectomy with an inguinal incision. The histopathology report revealed the diagnosis of testicular chloroma (Figure 1). The bone marrow aspiration and biopsy did not reveal the presence of any blast cell infiltration. The bone marrow cytogenetic analysis revealed only a 15% loss of chromosome Y. Staging with abdominal computerized tomography (CT) scan demonstrated a solid 4 cm lesion in the right paraaortic area (Figure 2), in proximity to the inferior vena cava and the right ureter causing hydronephrosis, for which a JJ ureteric stent was inserted. 

The multidisciplinary (MDT) meeting suggested intravenous chemotherapy with an induction therapy of Idarubicin (12 mg/m^2^ day 1, day 3), plus continuous infusion of Cytarabine 200 mg/m^2^ days 1-5 (IDA/Ara-C 2 + 5 regimen) and a prophylactic intrathecal chemotherapy of 100mg Cytarabine on day 3 (cerebrospinal fluid examination was normal). The CT scan for restaging demonstrated a partial remission of the right paraaortic lesion (2 cm in width) and the JJ stent was removed. A further induction-consolidation cycle of high-dose Ara-C followed. Thereafter, the CT scan demonstrated a complete remission of the right paraaortic lesion.

During the follow-up, after ten months, a metachronous mass at the left testis was revealed on US. Left radical orchiectomy, with an inguinal incision, was performed and the histopathology report demonstrated again the diagnosis of chloroma. Restaging with CT scan was negative and cycle of chemotherapy with the high-dose Ara-C regimen was performed. Six months after the second radical orchiectomy, the patient is recurrence-free.

## 3. Discussion

Chloroma is equivalent to extramedullary AML and is usually concomitant with the diagnosis of AML (1-4). It can also present as an unusual recurrence of AML in the patients who were initially diagnosed with AML and had achieved remission with chemotherapy. More rarely the diagnosis of chloroma can precede the diagnosis of full-blown AML by a few months or even years. Therefore, chloroma is regarded as an oncologic entity equivalent to the classic AML and is treated as such, with induction chemotherapy regimens, followed by cycles of consolidation chemotherapy. The objective is to achieve disease eradication and prevent recurrence, which unfortunately occurred in our case with the appearance of metachronous testicular chloroma.

In our rare case, except intravenous chemotherapy the patient also received intrathecal (in order to cross the blood-brain barrier) chemotherapy with 100 mg of Cytarabine (adjusted to patient’s age) at the time of the induction chemotherapy. This was performed because testicular infiltration by the malignant hemopoietic neoplasm can be complicated by central nervous system involvement, based on relevant experience regarding lymphoid malignancies (i.e. especially acute lymphoblastic leukemia and aggressive B-cell lymphomas) (5). After the high-dose Ara-C consolidation cycle, our patient’s CT scan demonstrated complete remission of the paraaortic mass.

During the MDT meeting we discussed with the radiotherapists the option to treat our patient with prophylactic radiotherapy at the contralateral testis as well as the possibility of consolidation radiotherapy to the region of the aforementioned right paraaortic mass. The presentation of the metachronous testicular chloroma did not justify our decision not to proceed with prophylactic radiotherapy at the contralateral testis. As relevant literature lacks, based on our experience we suggest phophylactic radiotherapy at the normal testis in cases of testicular chloroma (6).

In conclusion, in testicular chloroma intravenous and intrathecal chemotherapy, (induction and consolidation) is advisable as well as phropylactic radiotherapy at the contralateral testis. Close follow-up for possible recurrence and development of full-blown AML is strongly suggested.

## References

[1] Vardiman JW, Thiele J, Arber DA, Brunning RD, Borowitz MJ, Porwit A (2009). The 2008 revision of the World Health Organization (WHO) classification of myeloid neoplasms and acute leukemia: rationale and important changes.. Blood..

[2] Lagerveld BW, Wauters CA, Karthaus HF (2005). Testicular granulocytic sarcoma without systemic leukemia.. Urol Int..

[3] Pileri SA, Ascani S, Cox MC, Campidelli C, Bacci F, Piccioli M (2007). Myeloid sarcoma: clinico-pathologic, phenotypic and cytogenetic analysis of 92 adult patients.. Leukemia..

[4] Constantinou J, Nitkunan T, Al-Izzi M, McNicholas TA (2004). Testicular granulocytic sarcoma, a source of diagnostic confusion.. Urology..

[5] Avni B, Rund D, Levin M, Grisariu S, Ben-Yehuda D, Bar-Cohen S (2012). Clinical implications of acute myeloid leukemia presenting as myeloid sarcoma.. Hematol Oncol..

[6] Tsimberidou AM, Kantarjian HM, Estey E, Cortes JE, Verstovsek S, Faderl S (2003). Outcome in patients with nonleukemic granulocytic sarcoma treated with chemotherapy with or without radiotherapy.. Leukemia..

